# The Poisons and Pharmacy Bill

**Published:** 1908-08-22

**Authors:** 


### The Poisons and Pharmacy Bill.

This Bill has approached a stage nearer being an Act of Parliament, and it has received some further amendments at the hands of the House of Lords. Two of these are of special interest and value, the first concerning the title Pharmacist, the second relating to alterations in the Schedule of Poisons. The Bill is of most direct concern to those who are at present termed chemists and druggists, and it has been very fully criticised from time to time in the *Pharmaceutical Journal*; medical men, however, will be interested in the two points just mentioned. The title "chemist" has always been confusing; for although the context will usually make it clear whether an apothecary or an analytical chemist is meant by the term, and although the word chemist has now come to be more often used of a druggist than of an analyst, it is clear that in its original meaning this is not right. The terms "chemist and druggist" or "dispensing chemist" define their own meaning more obviously than does plain "chemist," but unfortunately even these have been permitted to drift a good deal away from their original meaning; first, because a certain number of duly registered chemists and druggists have allowed their statutory designation to become descriptive of a business which is sometimes of so miscellaneous a character that dispensing and dealing with medicines in the dispensing sense have become almost a minor part of it; secondly, because non-qualified persons, by forming themselves into a company may employ dispensers, and so forth, and behave as so-called chemists and druggists, apparently without the individuals who form the company being qualified as "chemists" at all. The amendment to the new Act proposes to remedy this by permitting all persons who are registered under the Pharmacy Acts to use the title "pharmacist," and it seems fairly clear that it is intended to prohibit the use of this title by bodies corporate of every description. The effect of this cannot but be good. The title "pharmacist" is the single word which, better than all others, describes the legitimate business of a dispensing chemist and druggist. It is a term which at present has no second meaning. It is not ambiguous. One effect, therefore, of the Poisons and Pharmacy Bill, if it becomes law, will be to prevent the further unchecked assumption and use of the description "chemist and druggist," whilst another effect will be to reserve to duly qualified *individuals* one very distinctive title which no one but a duly qualified person will be permitted to use. The other important amendment made by the House of Lords is in connection with the Schedule of Poisons. At present this Schedule can only be altered by addition—that is to say, a poison that is not in the Schedule may be added to it, but no poison at present in the Schedule can be removed from it or re-arranged in it. The new amendment provides that the Schedule may also be amended by deletion, or by transference of an included article from one part of it to another. As the *Pharmaceutical Journal* points out this is a matter of great importance; for, with this provision in the Act, it will be a matter of secondary importance whether the Schedule of Poisons as made by Parliament is perfect or not, since it will not be a very difficult matter to amend the Schedule should experience indicate any necessity for amendment. At the same time the new Schedule of Poisons has been arranged so much more clearly than the old, that little alteration will probably be required in it, at any rate for some time to come.

